# Single-dose Tamsulosin Induces Reversible Azoospermia and Ejaculatory Dysfunction Suggesting Potential for on-demand Male Contraception

**DOI:** 10.1590/S1677-5538.IBJU.2025.0601

**Published:** 2026-02-20

**Authors:** Leonardo Seligra Lopes, Julia Domingues Candelaria, Felipe Placco Araujo Glina, Thais Ventura Feitosa, Bruna Bizio Parra de Oliveira, Willy Roberto Camargo Baccaglini, Erik Montagna, Caio Parente Barbosa, Jose de Bessa, Sidney Glina

**Affiliations:** 1 Disciplina de Urologia do Centro Universitário FMABC Santo André SP Brasil Disciplina de Urologia do Centro Universitário FMABC, Santo André, SP, Brasil; 2 Instituto Ideia Fertil Santo André SP Brasil Instituto Ideia Fertil, Santo André, SP, Brasil; 3 Departamento de Cirurgia da Universidade Estadual de Feira de Santana Feira de Santana BA Brasil Departamento de Cirurgia da Universidade Estadual de Feira de Santana, Feira de Santana, BA, Brasil

**Keywords:** Ejaculatory Dysfunction, Azoospermia, Receptors, Adrenergic, alpha

## Abstract

**Purpose:**

Ejaculatory alterations are among the most frequent sexual side effects of α_1_-adrenergic antagonists. Although often attributed to retrograde ejaculation, recent evidence indicates that tamsulosin primarily disrupts seminal emission, occasionally leading to transient azoospermia. This study evaluated the frequency, timing, and reversibility of ejaculatory and seminal changes following a single oral dose of 0.8 mg tamsulosin in healthy men.

**Materials and Methods:**

Thirty-one healthy male volunteers (aged 18–45 years) underwent a baseline semen analysis, followed by six additional collections at 1–3 week intervals. Each collection was performed at a different post-dose time point, spaced every 4 hours, to construct a 24-hour post-administration profile. Semen parameters were assessed according to WHO criteria, and post-ejaculatory urine was examined to detect retrograde ejaculation. Temporal variations were analyzed using repeated-measures ANOVA, with effect sizes estimated by Cohen's d.

**Results:**

Seminal volume decreased significantly in 93.6% of participants, with aspermia in 80.7%, peaking 12 h after ingestion (p<0.001, d=2.05). Sperm concentration declined markedly, with azoospermia in 80.7% (p<0.001, d=1.59) and normalized after wash-out in 2 days. No retrograde ejaculation was observed. Adverse effects were mild and self-limited. A single 0.8 mg dose of tamsulosin caused a consistent, time-dependent disruption of seminal emission, producing transient azoospermia rather than retrograde ejaculation.

**Conclusions:**

A single 0.8 mg dose of tamsulosin transiently suppressed seminal emission, leading to reversible azoospermia within 12 hours most recovered by 24h, and all recovered within 48h. Its predictable, reversible effect supports caution in men seeking conception and further exploration as an on-demand male contraceptive model.

## INTRODUCTION

Tamsulosin is a type of alpha-blocker that selectively targets α_1_-adrenergic receptors. It helps relieve lower urinary tract symptoms caused by benign prostatic hyperplasia (BPH) and can also be used alongside other treatments to aid in passing ureteral stones (1, 2). Beyond its therapeutic role, alpha-blockers are known to cause ejaculatory disorders (1, 3), that vary among various drugs and are more significant depending on the dose used, which was classically interpreted as retrograde ejaculation. (1, 3–7). However, accumulating experimental and clinical evidence suggests that these events result instead from failure of seminal emission, leading to aspermia or even transient azoospermia (5, 8, 9).

Experimental models demonstrate that α_1_-receptors mediate contractility of the vas deferens, seminal vesicles, and epididymal tail (8, 10–14). Pharmacological blockades therefore disrupt coordinated emissions and prevent seminal fluid and sperm expulsion (9, 12, 15). Clinically, this results in anejaculation, markedly reduced volume, and sometimes complete absence of sperm in the ejaculate (9, 16).

The present study aimed to characterize the time course and reversibility of ejaculatory emission failure and azoospermia following a single 0.8 mg oral dose of tamsulosin in healthy volunteers, and to confirm whether these alterations are due to true emission failure rather than retrograde ejaculation.

## MATERIALS AND METHODS

Male volunteers were recruited based on the following inclusion criteria: age between 18 and 45 years, lack of continuous alpha-blocker medication, and the ability to collect sperm by masturbation. The exclusion criteria were history of neurological disorders, liver disease, nephropathy, use of exogenous testosterone, anabolic or androgenic steroids, diabetes mellitus, pelvic or genital surgery, concomitant use of specific medications (ketoconazole, erythromycin, other alpha-blockers, warfarin, and diclofenac), altered sperm analysis according to WHO standards (17), and refusal to provide an informed consent form (ICF).

Individuals were clinically assessed for anatomical alterations in the genitourinary tract or neurological alterations associated with ejaculatory dysfunction risk within specific physical exam. An initial semen sample was collected to establish the baseline parameters for study inclusion and to serve as a control without the use of medication.

The medications should be taken using a commercial presentation of tamsulosin hydrochloride (OmnicOcas ® Astellas Pharma Europe B.V., Hogemaat 2, 7942 JG Meppel, The Netherlands, registered and imported by *Astellas Farma Brasil Importação e Distribuição de Medicamentos Ltda*, São Paulo, SP, Brazil) of 0.4 mg, and orally administered two tablets in a single dose totaling 0.8 mg under controlled ingestion, guided by a single evaluator, and reinforced by telephone. The administration time of medication was standardized to align with morning semen analysis. A 0.8 mg dose was chosen in accordance with previous studies reporting a more pronounced influence on ejaculatory dynamics and semen analysis findings compared with lower doses (10, 18).

**Figure 1:**
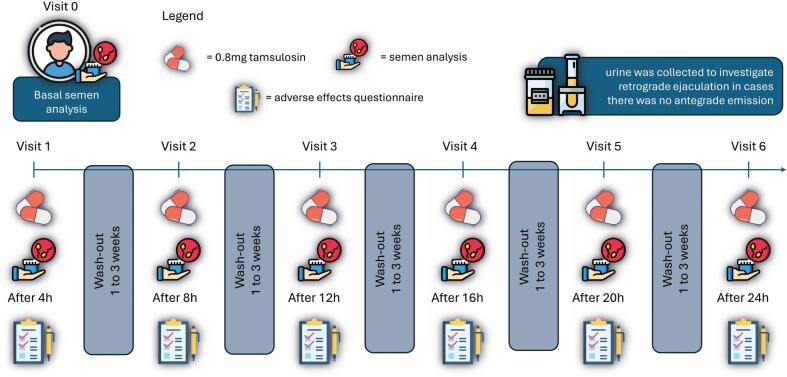
Study flow and medication schedule with subsequent semen analysis

Each collection was performed at a different post-dose time point, spaced every 4 hours (h), to construct a 24-h post-administration profile. All samples were collected by masturbation in a sterile collection bottle. The analyses were performed by experienced professionals from an assisted reproduction clinic under WHO recommendations (17), including a 2–3-day abstinence period and regulated quality standards, including centrifugation of the sample when no sperm was found in the initial evaluation. However, for statistical analysis, seminal volume and sperm concentration were considered. The individuals had their semen samples collected by masturbation, and immediately afterwards, their urine was collected to investigate retrograde ejaculation in cases where there was no antegrade emission or low volume. Urine was collected in a sterile vial containing 1 mL buffered culture medium (Figure-1, study design protocol).

The participants were questioned on their return visits about the potential adverse effects of single-dose alpha-blocker use.

The sample size was determined based on previously reported semen volumes and sperm concentration values after alpha-blockers oral intake (10, 16). The highest calculated values were obtained. Accordingly, to ensure statistical significance with a 95% confidence interval, a minimum of 14 participants was required. When limiting Type II errors with a statistical power of 0.8, the total number of participants required increased to 28.

We assumed that a reduction of > 95% would be considered clinically significant for changes in either volume or concentration. The variability in semen volume and sperm concentration is well recognized because of the intrinsic biological nature of spermatogenesis, which may exhibit high variation in response to different stimuli and conditions (17). Therefore, a sample size of 28 participants was selected to accommodate natural fluctuations and biological effects.

Categorical variables are expressed as relative and absolute frequencies. To compare the variation in the measurements, we used the repeated measures ANOVA test and post-hoc analysis with paired comparisons to identify the specific and significant moments of difference between the intervals, as well as the Benjamini-Hochberg correction to adjust the p-values and balance the risk of false positives. To assess the magnitude of the effect on ejaculatory volume and seminal concentration, we used the calculation proposed by Cohen, which is the difference between the means of the two groups divided by their joint standard deviation. The interpretation of Cohen's d depends on the values obtained: values less than or equal to 0.2 have a small effect, between 0.3 and 0.7, a medium effect and from 0.8 onwards, the effect is considered large. Phyton ® version 3.9, and Jupyter Notebook version 7.0.8 software were used.

This study was approved by the local ethics committee under protocol CAEE26124719.2.0000.0082 of May 2021 and was registered in the Brazilian Registry of Clinical Trials under number RBR-3hsj6g6.

## RESULTS

Thirty-four individuals (mean age 24.45 years, SD 4.71) who met the inclusion criteria were recruited between December 2021 and January 2023. Two individuals were requested to leave the study because they were unable to complete the collection period adequately. One patient requested to leave owing to mild adverse effects, as described later. Thirty-one patients completed the study. Tables 1 and 2 show the characteristics of the recruited individuals and baseline seminal volume and concentration values.

**Table 1:** Characteristics of the study population.

Characteristics		n	%
Participants		31	100
**Smoking**			
	Yes	3	09.68
	No	28	90.32
**Alcohol consumption**			
	Yes	15	48.39
	No	16	51.61
Use of medication*			
	Yes	7	22.58
	No	24	77.42

* not described as related to seminal alteration or interaction with tamsulosin.

**Table 2:** Seminal analysis baseline values.

Variables	Median	IQR	p*
Basal volume (mL)	3	2.25-4.0	0.024
Basal concentration (million/mL)	134.2	85.85-199.55	0.002

* Shapiro-Wilk test

**Figure 2:**
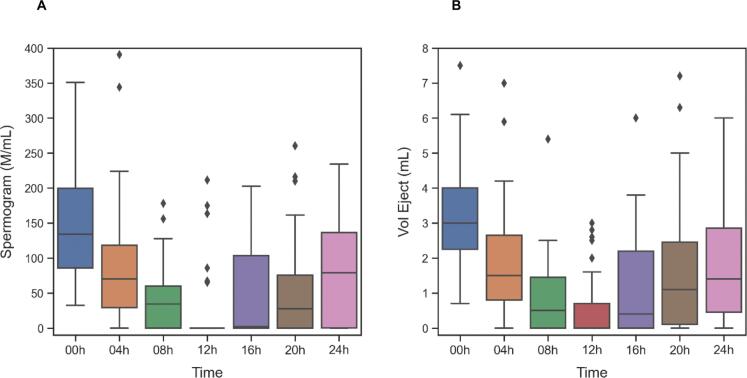
Variation in seminal volume and sperm concentration over 24 hours at intervals of every 4 hours after taking a single dose of tamsulosin 0.8mg.

**Figure 3:**
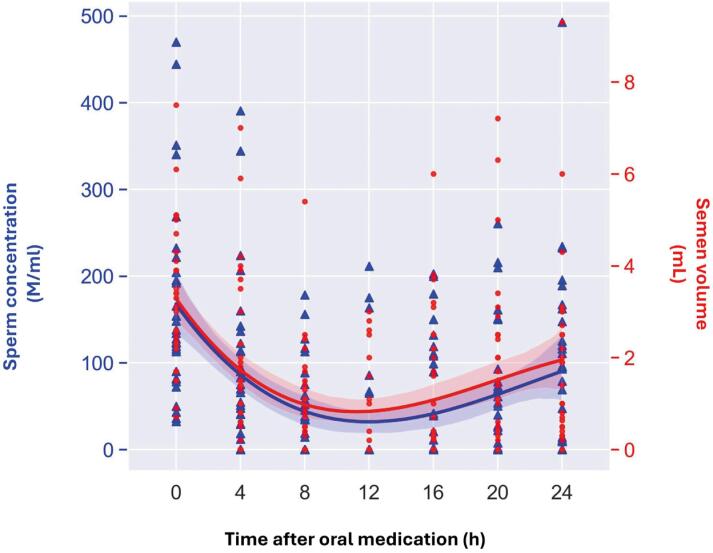
Temporal variation in sperm concentration and seminal volume following a single 0.8 mg dose of tamsulosin.

After all seminal analyses during the determined periods, a decrease in seminal volume was identified in 29/31 (93.6%) individuals, of which 25/31 (80.7%) had aspermia (Table-3A). In relation to sperm concentration, values below the 5th percentile of the WHO standard (17), considered oligozoospermia and/or azoospermia, were observed in 27/31 (87.1%) individuals. Of these, 25/31 (80.7%) presented azoospermia in at least one sample (Table-3B). Urine analyses confirmed the absence of spermatozoa, excluding retrograde ejaculation. Figure-2 demonstrates variation in seminal volume and sperm concentration over 24h. The decrease and subsequent recovery in seminal volume occurred concomitantly with changes in sperm concentration, indicating an effect throughout the entire ejaculatory pathway, from the vas deferens to the seminal vesicles. (Figure-3, scatter plot with smoothed regression lines).

**Table 3:** Evaluation of seminal volume(3A) and sperm concentration (3B) in relation to the interval in hours after ingestion of tamsulosin 0.8mg.

3A	Collection interval (h)	Median [IQR]	hypospermia n (%)*	aspermia n (%)*
**Seminal volume (mL)**	Baseline	3.0 [2.25-4.0]	0 (0)	0 (0)
	4	1.5 [0.8-2.65]	12 (38.7)	2 (6.4)
	8	0.5 [0.0-1.45]	10 (32.2)	11 (35.4)
	12	0.0 [0.0-0.7]	4 (12.9)	21 (67.7)
	16	0.4 [0.0-2.2]	8 (25.8)	10 (32.2)
	20	1.1 [0.1-2.45]	9 (29.0)	8 (25.8)
	24	1.4 [0.45-2.85]	4 (12.9)	3 (9.6)
**Number of individuals affected**			29/31 (93.6)	25/31 (80.65)
**3B**	Collection interval (h)	Median [IQR]	Sperm concentration lower than WHO p5 n (%)*	azoospermia n (%)*
**Sperm concentration (M/mL)**	Baseline	134.2 [85.85-199.55]	0 (0)	0 (0)
	4	70.0 [29.3-118.25]	6 (19.35)	5 (16.1)
	8	34.3 [0.0-59.85]	14 (45.16)	13 (41.9)
	12	0.0 [0.0-0.0]	25 (80.65)	25 (80.65)
	16	2.0 [0.0-103.4]	17 (54.83)	15 (48.4)
	20	27.5 [0.0-75.55]	13 (41.93)	11 (35.48)
	24	79.0 [0.24-136.45]	13 (41.93)	7 (22.58)
**Number of individuals affected**			27/31 (87.1)	25/31 (80.7)

* Number of individuals in the period (there may be repetitions in the intervals)

A – Box plot of the median variation and 95% confidence interval of sperm concentration at each collection time; M/mL = Million/mL; B = Box plot of the median variation and 95% confidence interval of seminal volume at each collection time; mL = mililiter.

M = Million; mL = milliliter; h = hours. Blue triangles represent individual sperm concentration values (left y-axis) and red circles represent seminal volume measurements (right y-axis) obtained at sequential post-dose time points. Smoothed regression lines illustrate the parallel decline and subsequent recovery of both parameters.

Repeated-measures analysis of variance (repeated-measures ANOVA) was performed to assess differences in seminal parameters over different collection times. To identify pairs of moments with significant differences, we performed paired multiple comparison analysis (post-hoc analysis) (Table-4).

**Table 4:** Analysis of the time of change in seminal parameters in relation to the tamsulosin 0.8mg intake interval.

Variable	Collection interval (h)	Mean	SD	p*	p cor**	d***
**Seminal volume (mL)**	Baseline	3.20	1.53			
	4	1.96	1.71	0.003	0.007	0.760
	8	0.95	1.17	< 0.001	< 0.001	1.647
	12	0.55	0.99	< 0.001	< 0.001	2.049
	16	1.23	1.54	< 0.001	< 0.001	1.282
	20	1.60	1.87	< 0.001	< 0.001	0.936
	24	1.89	1.99	0.001	0.003	0.734
**Sperm concentration (M/mL)**	Baseline	165.84	111.27			
	4	91.88	94.53	0,005	0,012	0.716
	8	41.50	50.96	< 0.001	< 0.001	1.437
	12	24.77	57.47	< 0.001	< 0.001	1.593
	16	51.41	68.02	< 0.001	< 0.001	1.241
	20	58.77	74.22	0.001	0.003	1.132
	24	90,91	106.79	0.012	0.023	0.687

* ANOVA of repeated measures;

** post-hoc analysis with paired comparisons;

*** Cohen's d effect size. mL = mililiter; M/mL = million/mL

A sharp decline in seminal volume was observed as early as 4h post-dose and also there was a marked reduction in seminal volume during the first 12h, with the lowest average volume recorded during this period (0.55 mL). Sperm concentration also decreased significantly after the initial collection and then began to increase gradually, as with seminal volume. The reduction in concentration was substantial at 8h and 12h (d = 1.437 and 1.593, respectively), representing large effects.

**Figure 4:**
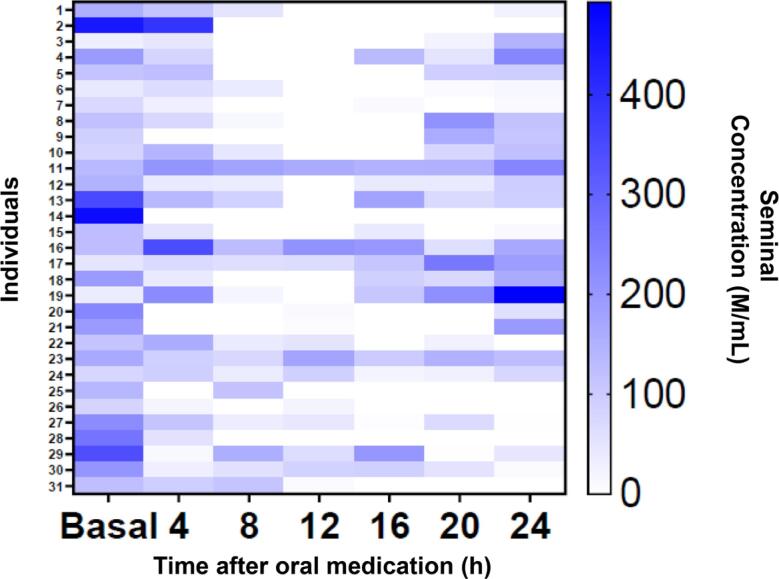
- Heat map showing individuals sperm concentration.

At 24h post-dose 7 individuals (22.6%) remained with azoospermia, however all of them returned to antegrade ejaculation with spermatozoa confirmed with a sperm analysis 2 days after last oral medication (Figure-4, heat map showing individuals sperm concentration).

M/mL = Million/miliLiter; h = hours.

Adverse effects were mild (headache, dizziness, rhinitis), and no systemic intolerance occurred. No participant experienced nausea, syncope, or clinically significant hemodynamic changes (Table-5). One participant requested to leave the study because of diarrhea (one episode of liquid stool) within 48 hours of taking the medication.

**Table 5:** Occurrence of adverse effects potentially related to the use of oral tamsulosin 0.8mg single dose.

Symptoms	4h n (%)	8h n (%)	12h n (%)	16h n (%)	20h n (%)	24h n (%)
Dizziness	0 (0)	4 (12.9)	3 (9.6)	1 (3.2)	2 (6.4)	1 (3.2)
Headache	4 (12.9)	4 (12.9)	8 (25.8)	5 (16.1)	4 (12.9)	3 (9.6)
Palpitation	1 (3.2)	0 (0)	2 (6.4)	1 (3.2)	2 (6.4)	1 (3.2)
Postural hypotension	0 (0)	0 (0)	2 (6.4)	0 (0)	6 (19.4)	0 (0)
Rhinitis	0 (0)	5 (16.1)	7 (22.6)	5 (16.1)	0 (0)	6 (19.4)
Constipation	0 (0)	1 (3.2)	2 (6.4)	0 (0)	0 (0)	2 (6.4)
Diarrhoea	0 (0)	0 (0)	0 (0)	1 (3.2)	0 (0)	0 (0)
Skin rash	0 (0)	0 (0)	1 (3.2)	0 (0)	0 (0)	0 (0)
Itching	0 (0)	0 (0)	1 (3.2)	0 (0)	0 (0)	0 (0)
Weakness	0 (0)	0 (0)	1 (3.2)	1 (3.2)	0 (0)	0 (0)

## DISCUSSION

A single 0.8 mg dose of tamsulosin produced a consistent, time-dependent interruption of seminal emission leading to azoospermia in over 80% of healthy men. The magnitude and reversibility of these findings reinforce the concept that selective α_1_-adrenergic blockade impairs emission rather than ejaculation per se.

Animal studies indicate that alpha1A-adrenergic receptors mediate contraction of the cauda epididymis (11), vas deferens (12, 19), and seminal vesicles (12) in rats and pigs, whereas alpha1D-adrenergic receptors affects (9) the contraction of both the epididymis and vas deferens when induced by noradrenaline, contributing to ejaculation disorder by altering the sperm emission phase. (19) A comparative study of four different types of alpha-blockers in rats revealed increased seminal vesicle dimensions and reduced ejaculatory function, with the effects depending on the type of medication and dose. (5) Studies in pigs have evaluated the role of both alpha- and beta-adrenergic receptors in response to noradrenaline stimulation in electrical impulses and the antagonistic effect of the use of beta- and alpha-blockers. (13, 14)

In clinical trials ejaculatory disorders were more prevalent with alpha-blockers than placebo (OR 5.88; p<0.0001), particularly selective alpha1A blockers such as tamsulosin (OR 8.58; p=0.006) and silodosin (OR 32.5; p<0.0001). (6) Ejaculation effects can be reduced by adjusting the dosage and administration of tamsulosin (20, 21). In a study involving 1,740 men taking 0.4 mg of tamsulosin continuously for LUTS(21), 6.7% reported ejaculatory disorders and were randomized to reduce the dose to 0.2 mg, take 0.4 mg every other day or maintain the dosage for 3 months. The response rates for symptom maintenance were 9.6, 25.8, and 100%, respectively.

Differentiating between anejaculation and retrograde ejaculation is clinically relevant because anejaculation results in azoospermia. This differentiation, based solely on an individual's clinical complaint, can lead to under-diagnosis; therefore, it should ideally involve sperm testing in the urine after ejaculation (22). Grasso et al. (23) found sperm in the urine post-ejaculation in 6 of 10 individuals taking tamsulosin 0.4 mg for BPH who complained of decreased seminal volume. In another study of 42 individuals taking either 0.4 mg tamsulosin or 0.8 mg silodosin for 12 weeks for BPH, all reported changes in ejaculation after starting the medication, and 66.7% had no sperm in their urine (22).

A previous study on the effects of alpha-blockers on seminal parameters in healthy adult men showed a reduction in seminal volume and sperm concentration in a group that used tamsulosin, with five individual presenting with azoospermia; none had sperm in the urine after ejaculation. (8) In one individual with marked hypospermia, transrectal ultrasound revealed reduced sperm emission and turbulence in ejaculatory ducts.

However, in a double-blinded, randomized, placebo-controlled pilot study, 57 individuals using medication for a period of 5 days were randomized into 3 groups (placebo, tamsulosin and alfuzosin) with 90% of the individuals using tamsulosin showing a decrease in seminal volume, and a significantly greater change in volume was found in the tamsulosin group (-2.4±0.17 mL) compared to placebo (+0.4±0.18, p<0.0001) or alfuzosin (+0.3±0.18, p<0.001) (25). A total of 35.4% of individuals with anejaculation were identified in the tamsulosin group, with no change in the presence of sperm in the urine after orgasm compared to baseline and no difference with placebo (18). In the present study, of 29/31 (93.6%) patients showed a change in volume, identifying 80.7% of individuals with anejaculation. In addition, 25 (80.7%) patients had no spermatozoa detected in post-ejaculatory urine. The higher incidence of aspermia observed in this study compared to previous reports is related to the use of doses higher than those typically prescribed in clinical practice. Moreover, ejaculation was documented during masturbation, whereas in real-life settings patients may not notice seminal emission if they engage exclusively in penetrative sexual activity.

The presence of beta-adrenergic receptors 1, 2, or 3 in the prostate has been described in a few studies, and, the main action is related to Beta-2 adrenergic receptors, and their blockade results in tissue contraction, particularly in individuals without BPH (24). This could explain the inconsistency in the effect of reducing sperm volume and concentration with the use of alpha-blockers in the two individuals in this study, who were taking beta-blockers continuously because of systemic arterial hypertension.

The theory of possible male contraception which involves blocking alpha1A-adrenergic action is also based on animal studies. Administration of tamsulosin to rats reduced the number of spermatozoa in the ejaculate as well as the number of embryos per pregnancy (25). In humans, Wang et al. (10) evaluated 40 young men aged 22 to 31 in a randomized, crossover, placebo-controlled study with a single dose of 0.4 mg and 0.8 mg tamsulosin and analyzed the seminal profile and urine after orgasm 4-6 hours after taking the medication. Anejaculation and azoospermia were found in 100% of the individuals who used the 0.8 mg dose, with a decrease in seminal volume and total mobile sperm count when the 0.4 mg dose was used (10). In the present study, we observed anejaculation in 80.7% of the patients, all of whom were diagnosed with azoospermia using the 0.8 mg single dose. Using silodosin, a selective alpha-blocker, Kobayashi et al. (26) evaluated 15 volunteer urologists in a double-blind study using a dose of 4 mg twice daily for 6 days, which was compared to a placebo. The authors observed anejaculation in all individuals, with absolute azoospermia confirmed using post-orgasm urine analysis. More recently, Bhat and Shastry (27) prospectively evaluated a cohort of 63 men with normal sperm counts and partners of reproductive age for one year. Silodosin 8 mg was administered approximately 3 hours before sexual intercourse. No unintended pregnancies have been reported. In all cases, azoospermia was reversible after discontinuation of the alpha-blocker treatment.

Although this study was not designed to assess contraception, the consistent and reversible induction of azoospermia supports the theoretical feasibility of α_1_ blockade as a non-hormonal approach to transient sperm suppression. The notion that selective α-blockers might act as reversible male contraceptives remains relevant, given accumulating evidence that men increasingly wish to share contraceptive responsibility with their partners (28). This has been a challenge for years, with most strategies using hormonal alternatives having undesirable side effects and/or long-term or irreversible effects on fertility (29). Because we did not evaluate repeated dosing, long-term use, pregnancy outcomes, or delayed doses, no contraceptive schedule or "safe window" can be proposed based on our findings. Because this was a mechanistic experiment, we selected the supratherapeutic dose to maximize detection of reversible ejaculatory blockade within a 24h window. The need for semen monitoring and contraceptive reliability requires dedicated larger and longer prospective studies.

An additional factor that may influence the magnitude and consistency of ejaculatory effects is the pharmaceutical formulation of tamsulosin. The oral controlled absorption system (OCAS) provides a slower and more uniform release profile, resulting in lower peak plasma concentrations and reduced fluctuations in systemic exposure compared with the immediate-release formulation (30). This pharmacokinetic behavior may attenuate the intensity of α_1_-adrenergic blockade at critical time points for seminal emission, potentially explaining the lower frequency or milder expression of ejaculatory dysfunction reported in some clinical series using OCAS formulations. Therefore, future studies aiming to precisely characterize the timing, reversibility, and magnitude of ejaculatory suppression should preferentially employ the immediate-release formulation of tamsulosin, allowing a clearer assessment of peak-related effects on seminal emission and facilitating comparison across pharmacodynamic endpoints.

The strengths of this study are represented by the standardized semen analysis, controlled timing of collections, objective urine analysis to rule out retrograde ejaculation, and paired intra-individual comparisons, guaranteeing the reversibility of the effect. The limitations include the small number of individuals in the sample despite the sample calculation and lack of hormonal assessment.

## CONCLUSION

In conclusion, a 0.8 mg dose of tamsulosin significantly reduced seminal volume and sperm concentration within the first 12h, often leading to azoospermia in numerous individuals. All individuals recovered from ejaculatory dysfunction with normal sperm concentration more than 24h post-medication, confirming the reversibility of the effect in a safe manner, with minimal adverse effects. Given its marked and reversible impact on seminal emission, tamsulosin should not be prescribed to men actively attempting conception, as many patients may not perceive the reduction in ejaculate volume during penetrative intercourse, potentially leading to unrecognized iatrogenic infertility. These findings also warrant further investigation of tamsulosin and related agents as potential pharmacologic models for on-demand male contraception.

## Data Availability

All data generated or analysed during this study are included in this published article
